# Strange Cause for Suicide!

**Published:** 1867-12

**Authors:** 


Strange Cause for Suicide!—That suicides have been committed for much worse reasons than are given by the actor in the following case, is no justification for his committing the rash act. We trust that none of our readers will study the relationship so deeply as to tempt them to follow the example of the unfortunate Titusville man.

"Some time since it was announced that a man at Titusville, Pennsylvania, committed suicide for the strange reason that he had discovered that he was his own grandfather. Leaving a dying statement, explaining this singular circumstance, we will not attempt to unravel it, but give his explanation of the mixed-up condition of his kinsfolk in his own words. He says: 'I married a widow who had a grown-up daughter. My father visited our house very often, fell in love with my step-daughter, and married her. So my father became my son-in-law, and my step-daughter my mother, because she was my father's wife. Some time afterward, my wife had a son; he was my father's brother-in-law, and my uncle, for he was the brother of my step-mother. My father's wife—*i.e.* my step-daughter had also a son; he was, of course, my brother, and in the meantime my grandchild, for he was the son of my daughter. My wife was my grandmother, because she was my mother's mother. I was my wife's husband and grandchild at the same time. And as the husband of a person's grandmother is his grandfather, I was my own grandfather."

